# Bayesian Analysis of Postoperative Complication Risk Associated With Preoperative Exposure to Fine Particulate Matter: A Single‐Center Cohort Study

**DOI:** 10.1111/aas.70235

**Published:** 2026-04-26

**Authors:** John F. Pearson, Cameron K. Jacobson, Calvin S. Riss, Matthew J. Strickland, Longyin Lee, Neng Wan, Tabitha M. Benney, Nathan L. Pace, Ben K. Goodrich, Jonah S. Gabry, John V. Pham, Cade K. Kartchner, Jacob A. Wood, Michael H. Andreae

**Affiliations:** ^1^ Department of Anesthesiology University of Utah School of Medicine Salt Lake City Utah USA; ^2^ Global Change and Sustainability Center, University of Utah Salt Lake City Utah USA; ^3^ Department of Anesthesiology, Perioperative and Pain Medicine Stanford University School of Medicine Stanford California USA; ^4^ Department of Biostatistics Epidemiology and Environmental Health, University of Nevada Reno School of Public Health Reno Nevada USA; ^5^ School of Environment, Society and Sustainability, University of Utah Salt Lake City Utah USA; ^6^ School of Public Affairs, University of Utah Salt Lake City Utah USA; ^7^ Applied Statistics Center, Columbia University New York City New York USA; ^8^ Indiana University School of Medicine Indianapolis Indiana USA

## Abstract

**Background:**

Air pollution, especially particle pollution, is increasingly recognized as a potential perioperative risk factor, yet modeling environmental exposures in surgical cohorts remains methodologically underdeveloped. We demonstrate a Bayesian hierarchical framework to quantify probabilistic associations between preoperative fine particulate matter (PM_2.5_) exposure and postoperative complications, highlighting its interpretability and flexibility for clinical environmental epidemiology.

**Methods:**

We conducted a single center, retrospective cohort study using data from 49,615 surgical patients in Utah who underwent elective surgical procedures from 2016 to 2018. Patients' addresses were geocoded and linked to daily Census‐tract level PM_2.5_ estimates. The exposure variable was defined as the maximum PM_2.5_ concentrations in the 7 days prior to surgery. The binary outcome was a composite of postoperative complications: pneumonia, surgical site infection, urinary tract infection, sepsis, stroke, myocardial infarction, or thromboembolic event. A hierarchical Bayesians regression model with weakly informative priors was used adjusting for age, sex, season, neighborhood disadvantage, and the Elixhauser index of comorbidities with census tract as a group (random) effect. We present posterior estimates with credible intervals, highlight model transparency and sensitivity, and discuss contrasts with standard frequentist methods.

**Results:**

Postoperative complications were associated in a dose‐dependent manner with higher concentrations of PM_2.5_ exposure. We found a relative increase of 8.2% in the odds of complications (OR = 1.082) for every 10.ug/m^3^ increase in the highest single‐day 24‐h PM_2.5_ exposure during the 7 days prior to surgery. For an increase in PM_2.5_ from 1 to 30 ug/m^3^, the odds of complication rose to over 27% (95% CI: 4%–55%). The results were robust across prior choices and model specifications. We report full posterior distributions and highlight advantages of Bayesian modeling for uncertainty quantification and clinical interpretability.

**Conclusions:**

This case study demonstrates the application of hierarchical Bayesian modeling to quantify the probabilistic associations between preoperative PM_2.5_ exposure and postoperative complications, highlighting transparent risk estimation and uncertainty characterization that may inform the design of future multicenter perioperative environmental studies.

**Editorial Comment:**

Using Bayesian statistical analysis, the authors demonstrate a dose‐dependent risk for postoperative complications in patients exposed to air polluted with fine particulate matter with a size of less than 2.5 μm.

## 1 Introduction

Short‐term exposure to fine particulate matter less than 2.5 μm in size (PM_2.5_) is a well‐established risk factor for cardiovascular, respiratory, and neurologic morbidity and mortality worldwide [1, 2, 3, 4, 5, 6]. Perioperative patients may be uniquely susceptible to PM_2.5_, as the physiological stress of surgery produces pulmonary trauma, hemodynamic stress, and proinflammatory cytokines [7, 8, 9, 10, 11, 12, 13], that overlap mechanistically with the inflammatory and thrombotic pathways triggered by air pollution exposure [14, 15]. This overlap suggests a potential interaction of surgical stress and acute PM_2.5_ exposure, yet the evidence remains limited.

Previous studies examining air pollution and perioperative outcomes are few, and often limited to specialized populations while almost exclusively using frequentist approaches [16, 17, 18, 19, 20]. These approaches provide limited capacity to communicate uncertainty in clinically interpretable probabilistic terms. Bayesian hierarchical methods offer an alternative: they allow integration of prior knowledge, direct probabilistic interpretation of effect sizes, and flexible handling of complex models. Despite these advantages, Bayesian approaches remain underutilized in perioperative environmental epidemiology.

**FIGURE 1:**
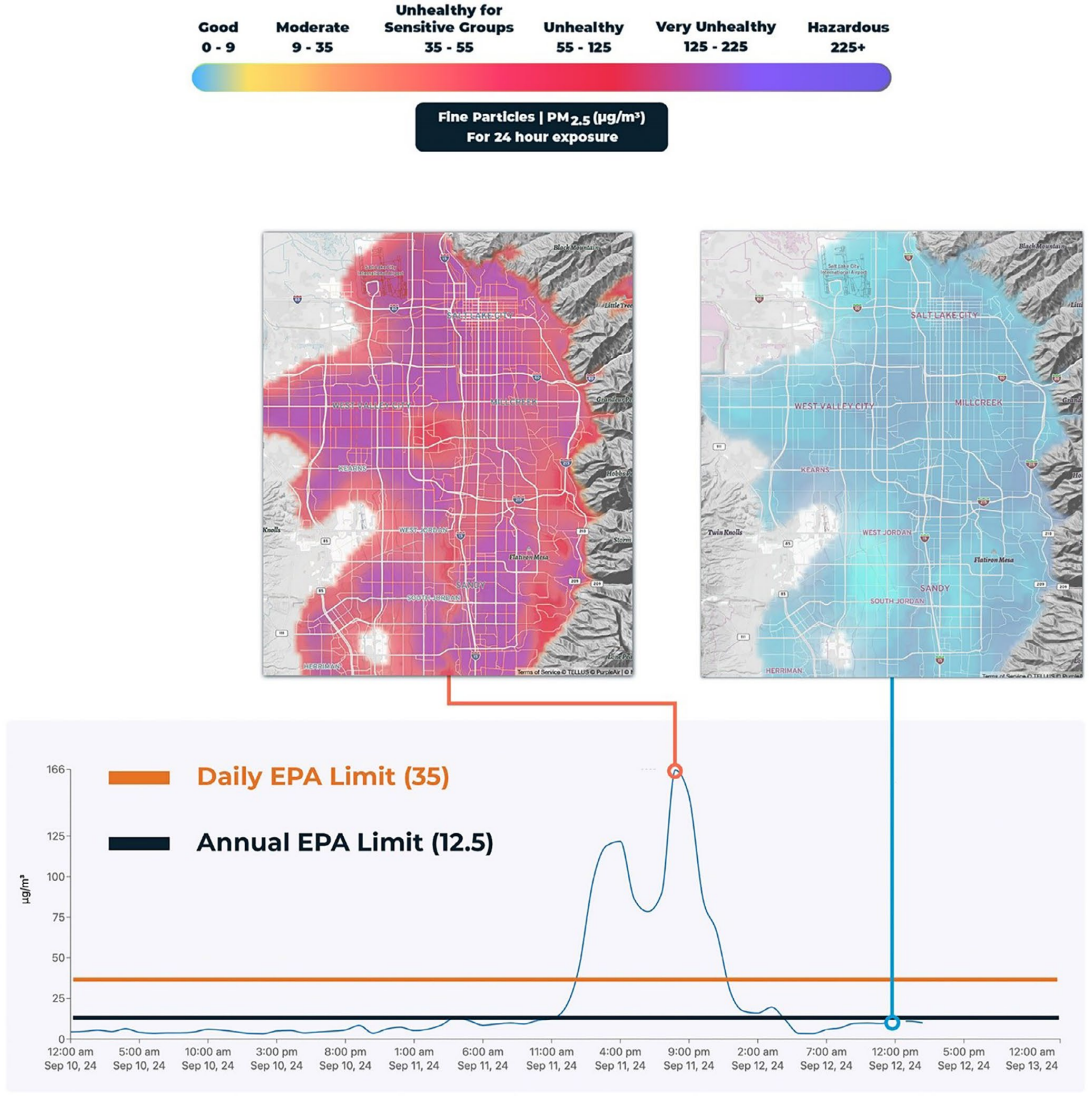
Rapidly changing pollution levels along the Wasatch Front. This figure illustrates the rapidly changing pollution levels along the “Wasatch Front” mountain range in Salt Lake City, Utah. The unique mountain geography of the “Wasatch Front,” along with five oil refineries, multiple industrial facilities, 2.2 million people, the intersection of two major interstate highways, and a national train depot work together to produce the increased occurrence of extreme pollution events in the region, which dramatically impact ambient PM_2.5_ concentrations [24, 46, 47]. This results in inversion conditions during the winter, with warm air aloft the valley trapping cold air and pollutants in the densely populated valley below. Similarly, in the summer when wildfires throughout the American West dominate pollution exposures, the geological bowl produced by the intersection of multiple mountain ranges that comprise the Wasatch Front act as a shield that accumulates wildfire smoke in the metropolitan region. In both instances, low pressure systems at random tend to rapidly clear the area of pollutants. As a result, patients are quasi‐randomly exposed to high or low pollution levels, while their surgery scheduling is disregarding this exposure, leading to a natural experiment. The left map illustrates high observed pollution levels (red/purple for unhealthy high pm 2.5 levels) due to a recent wildfire event on September 11–12, 2024 while the right map demonstrates how a change in weather patterns cleared the air, leading to suddenly much lower pollution exposures (blue). The graph demonstrates this timeline of rapidly changing 2.5 small particle pollution levels, with markers for the dates of the maps.

Northern Utah's Wasatch Front provides an ideal setting to explore acute pollution exposures. Wildfire smoke and winter inversions generate short‐lived but intense PM_2.5_ episodes, while elective surgery cases proceed independent of daily air quality (Figure 1: The rapidly changing pollution levels along the Wasatch Front). This setting enables a quasi‐natural experiment and an opportunity for a methodological case study of Bayesian modeling in a surgical cohort.

Therefore, our objective was two‐fold: first, to explore the relationship between preoperative PM_2.5_ and postoperative complications, in a large, single‐center cohort; and second, to demonstrate the feasibility and interpretive strengths of a hierarchical Bayesian approach for perioperative environmental health research. Therefore, we analyzed a composite complication outcome to enable an exploratory probabilistic evaluation of associations between preoperative PM_2.5_ exposure and postoperative complications, using Bayesian inference to emphasize interpretability, sensitivity to prior assumptions, and transparent communication of uncertainty.

## 2 Materials and Methods

### 2.1 Study Design and Data Sources

We performed a single center cohort analysis of the University of Utah local Multi‐Center Perioperative Outcomes Group (MPOG) [21] electronic health registry. This was supplemented with data from the University of Utah Health's electronic health record Epic (Epic Systems Corporation, Verona, Wisconsin, USA) database covering procedures from January 1, 2016 to December 31, 2018. We adhered to the Strengthening the Reporting of Observational Studies (STROBE) statement and principles [22]. The University of Utah Institutional Review Board approved the study under Exemption Category 4 (IRB #00142167). Prior to access of data, we documented our intent to examine the influence of environmental exposures on perioperative outcomes.

### 2.2 Study Population

**FIGURE 2:**
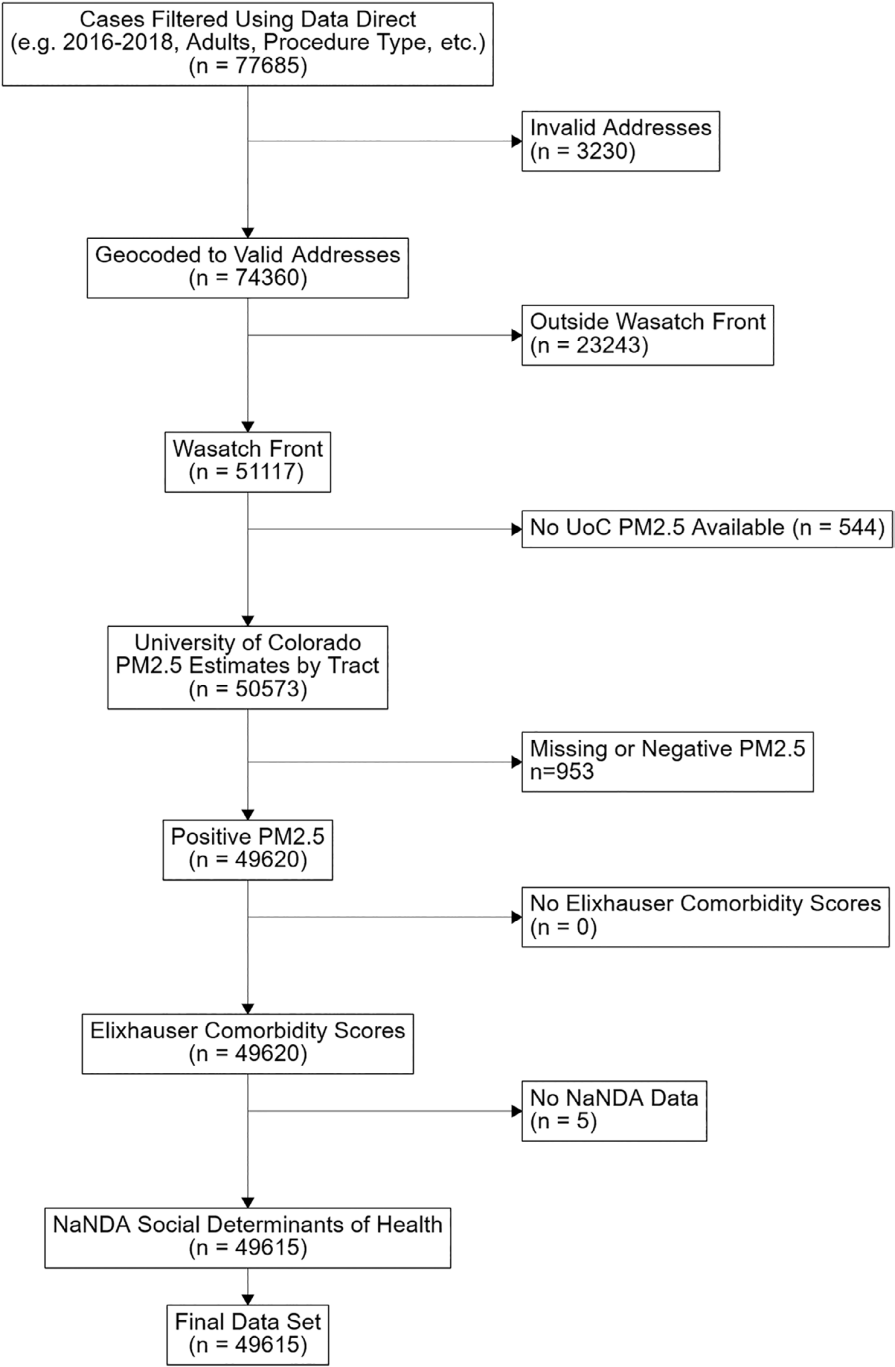
Consort flow diagram. This figure details our initial cohort downloaded from Data Direct from MPOG.org and the process of exclusion criteria applied to generate our final cohort. Note, the greatest number of patients eliminated was when excluding those who lived outside of the Wasatch Front study area.

We included elective and non‐emergent general anesthesia cases performed at University of Utah Health during the study period. We excluded cases with ASA 5–6, patients < 18 years of age, procedures performed without general anesthesia, and obstetric, electroconvulsive therapy, and bronchoscopy procedures. To focus on an exposure‐prone population, we limited the cohort to patients residing in Wasatch Front counties (Salt Lake, Utah, Davis, Weber, Cache, and Box Elder); areas that experience severe episodic air pollution from winter inversions and wildfire smoke [23, 24]. Addresses were geocoded to census tracts using ArcGIS Pro 3.0 and US Census TIGER/Line shapefiles. Patients were excluded if addresses were missing or invalid, if PM_2.5_ estimates were unavailable, or if the Elixhauser Comorbidity index could not be assigned (Figure 2). As this was a retrospective population‐based cohort, no a priori sample size calculation was performed. All eligible cases meeting inclusion criteria during the study period were analyzed to maximize statistical precision and stability of hierarchical Bayesian estimates.

### 2.3 Exposure: Fine Particulate Matter (PM_2.5_)

Daily mean PM_2.5_ estimates were derived from a machine learning model based on satellite, meteorological, and land‐use data [25] and assigned at the census tract of patient residential address at the time of surgery as recorded in Epic. For primary analysis, exposure was defined as the maximum concentration of PM_2.5_ in the 7 days prior to surgery (including the day of surgery as Lag0). This metric was chosen because (1) pollution events in northern Utah are typically of short duration [24], and (2) inflammatory and thrombotic effects of PM_2.5_ are most pronounced in the days immediately following exposure [26, 27].

### 2.4 Outcomes: Major Post‐Operative Complications

The primary outcome was a binary composite of major postoperative complications, identified from ICD‐10 coded discharge diagnoses in Epic and MPOG [28, 29, 30]. Therefore, all complications took place during the inpatient stay following the surgical procedure. The composite included pneumonia, surgical site infection, urinary tract infection, sepsis, stroke, myocardial infarction, or thromboembolic events. We classified the presence of any postoperative complication into a binary outcome measure, with a positive (yes) if any complication was present, while negative (no) if no complications were observed.

### 2.5 Covariates

Multivariable models were adjusted for patient age, sex, year, season, neighborhood deprivation score (from the National Neighborhood Data Archive incorporating poverty, race, education, and income [31]), and the Elixhauser comorbidity index [32, 33]. The Elixhauser comorbidity index was selected in place of the American Society of Anesthesiologists Physical Classification Score (ASA‐PS) because it has comparable predictive validity, is computable from administrative data, and avoids subjective assessment at the bedside [34, 35, 36]. We assigned the Elixhauser comorbidity index following standard assignment procedures [37]. We anticipated that complications would rise with increasing Elixhauser index. Procedure type and race/ethnicity were not included due to dataset limitations.

### 2.6 Statistical Analysis

#### 2.6.1 Plain Language Overview of Bayesian Modeling

In conventional (frequentist) regression, uncertainty is only expressed through repeated hypothetical sampling of the same population, and confidence intervals describe how the results would vary if the study were repeated many times. Essentially a frequentist approach says “Assuming the null hypothesis is true, what is the chance we would see results this or more extreme if we repeated this experiment over and over?” This contrasts with Bayesian regression, which treats the model parameters themselves as uncertain and estimates a probability distribution for each parameter given the observed data which is considered fixed. Bayesian analysis begins with a prior distribution, which represents reasonable assumptions about plausible effect sizes before examining the data, then updates this using observed data to produce a posterior distribution. This posterior distribution is a probability of the effect size, which then one can calculate credible intervals, the counterpart to confidence intervals in frequentist terms. The key difference is the Bayesian approach gives direct probabilistic interpretation, so that one can say “Given what we believed before, and given the data we actually observed, how *probable* is each possible effect size?” This lends itself better to clinical, bedside interpretation and risk communication.

#### 2.6.2 Primary Bayesian Model Specification

We fit a hierarchical Bayesian multivariable model to estimate the probabilistic associations between preoperative PM_2.5_ exposure and postoperative complications. The Season label was modeled categorically as “fire” (July–October), “inversion” (November–February), and “baseline” (March–June). Model fit was by hierarchical Bayesian regression methods using Markov chain Monte Carlo algorithms, specifically Hamiltonian Monte Carlo with the No‐U‐Turn Sampler having more rapid convergence for high‐dimensional models. Models were run with six chains and 2000 total iterations per chain, of which the first 1000 iterations were used for warm‐up (adaptation) and the remaining 1000 iterations per chain were retained as posterior samples.

Regression coefficients were assigned weakly informative priors with the normal prior (mean 0, SD = 1). This regularization improves stability and guards against overfitting hierarchical models with correlated covariates. For sensitivity analysis, the models were run with the R2D2M2 prior [38] *brms* M2 implementation (mean R2 = 0.25, precision = 4, *D* = 0.5). The R2D2 prior places a joint prior on the regression coefficient vector by directly specifying a prior on the model's expected coefficient of determination (R2). We specified R2 = 0.25 with precision = 4, *D* = 0.5, reflecting a conservative prior belief that only a small subset of predictors would have meaningful non‐zero effects, while inducing adaptive shrinkage toward zero for noise predictors.

For our model, we incorporated both PM_2.5_ and Neighborhood Disadvantage as a linear predictor, based in prior epidemiological literature demonstrating associations between adverse social determinants of health (e.g., poverty, income inequality) and increased air pollution exposure [39]. Conversely, we used a spline on Elixhauser and Age together, as our exploration of both suggested non‐linearity, and in surgical contexts, both patients and surgeons tend to defer high‐risk surgery at more advanced ages. Census tract was included in the statistical model as group (random) effect to account for spatial clustering and unmeasured neighborhood‐level confounding. Complete mathematical specification of the primary model, prior distributions, and full posterior summaries are provided in the Supporting Information. Convergence and sampling adequacy were assessed using the Gelman‐Rubin R‐hat statistic, effective sample size (ESS), chain mixing, and chain autocorrelation. All parameters achieved R‐hat = 1.00, bulk and tail ESS values for regressions parameter estimates exceeded 4000, and bulk and tail ESS values for spline and multilevel parameters exceeded 2000, which exceeded bulk and tail ESS of greater than 1000 recommended for stable estimation [40]. MSCE values were small relative to posterior standard deviations for all parameters. The posterior predictive distribution was used to generate a predictive accuracy metric as measured by leave‐one‐out cross‐validation. Nested models were compared by expected‐log‐predictive‐density. Model fits and parameter values were explored using conditional effects, *R* ^2^ coefficient of determination, and Bayesian hypothesis testing.

Model results are presented as parameter estimates (log odds ratio) using means, medians, standard deviations, and 95% credible intervals (CIs, using equal‐tailed‐percentile‐based); conditional effects were estimated to report and display the probability of complications. A 95% credible interval has a 95% probability of containing the true parameter value. Model coefficients are also presented with forest plots to show the probability of direction. Analyses were conducted using R Statistical Software v4.4.1, and used two software packages (brms, loo) built on STAN with a Hamiltonian Monte Carlo based software to estimate Bayesian models [41]. Additional data and model description was done in the R language using the tableone, loo, mcmcplot, posterior, and tidybayes packages. Sensitivity analyses evaluating prior robustness and numeral stability are reported in the Supporting Information.

## 3 Results

### 3.1 Study Population Characteristics

The initial dataset included 77,685 cases from January 1, 2016 to December 31, 2018. After exclusion for non‐Wasatch Front residence (*n* = 23,243), invalid addresses (*n* = 3230), and missing PM_2.5_ data (*n* = 544), the final analytic cohort was 49,615 patients. Median age was 53.2 years (SD = 17.7) and 52.2% were female. Comorbidity burden was low overall, with a mean Elixhauser of 0.89 (SD = 1.95). Most patients resided in Salt Lake County (*n* = 34,399), while annual case volume increased modestly from 15,375 in 2016 to 16,704 in 2018. Cohort characteristics are summarized by year in Table 1.

**TABLE 1:** Cohort characteristics by year.

Factor	Level	Overall	2016	2017	2018	SMD
Sample total		49,615	15,375 (30.9)	17,536 (35.3)	16,704 (33.6)	
Complications (%)	No	47,204 (95.1)	14,586 (94.9)	16,661 (95.0)	15,957 (95.5)	0.02
	Yes	2411 (4.8)	789 (5.1)	875 (5.0)	747 (4.5)	
Elix (mean [SD])		0.89 (1.95)	0.83 (1.83)	0.91 (2.02)	0.91 (1.99)	0.029
Sex (%)	Male	23,704 (47.8)	7339 (47.7)	8366 (47.7)	7999 (47.9)	0.002
	Female	25,911 (52.2)	8036 (52.3)	9170 (52.3)	8705 (52.1)	
Season (%)	None	17,044 (34.4)	4930 (32.1)	5980 (34.1)	6134 (36.7)	0.075
	Fire	16,472 (33.2)	5457 (35.5)	5704 (32.5)	5311 (31.8)	
	Cars	16,099 (32.4)	4988 (32.4)	5852 (33.4)	5259 (31.5)	
Disadvantage (mean [SD])		0.06 (0.04)	0.06 (0.04)	0.06 (0.04)	0.06 (0.04)	0.013
Age (mean [SD])		53.22 (17.6)	53.31 (17.61)	53.43 (17.73)	52.92 (17.67)	0.019
County name (%)	Box elder	672 (1.4)	193 (1.3)	246 (1.4)	233 (1.4)	0.034
	Cache	1098 (2.2)	335 (2.2)	386 (2.2)	377 (2.3)	
	Davis	7047 (14.2)	2055 (13.4)	2526 (14.4)	2466 (14.8)	
	Salt Lake	34,399 (69.3)	10,827 (70.4)	12,113 (69.1)	11,459 (68.6)	
	Utah	3847 (7.8)	1179 (7.7)	1386 (7.9)	1282 (7.7)	
	Weber	2552 (5.1)	786 (5.1)	879 (5.0)	887 (5.3)	

*Note:* Overall cohort is presented by year. Complications fell marginally year over year, while the overall number of cases remained relatively steady. The majority of cases were in Salt Lake County, where the greatest number of University of Utah operating rooms are located.

### 3.2 Post‐Operative Complications

The overall rate of the composite complication outcome was 4.85% (*n* = 2411 patients with events among total cohort of 49,615). The composite bundle consisted of pneumonia (*n* = 509), surgical site infection (*n* = 589), urinary tract infection (*n* = 739), sepsis (*n* = 1353), stroke (*n* = 1), myocardial infarction (*n* = 184), and thromboembolism (*n* = 431) (n.b. patients may have had more than one complication, but only counted for one in model). Rates varied by County, from 4.41% in Davis County to 7.58% in Box Elder County. Male patients experienced complications more often than females (5.94% vs. 3.86% respectively). Complication risk increased with Elixhauser comorbidity index: patients without complications having a mean index of 0.6 (SD = 1.52) compared with 5.15 (SD = 3.72) among those with complications. Complications did not vary by season. Cohort characteristics are summarized by presence of a complication in Table 2. When PM_2.5_ was dichotomized by the EPA daily limit of 35 ug/m^3^, complication rates increased from 4.8% below the threshold to 6.2% above. In the Bayesian model, the posterior probability of higher risk at exposures > 35 ug/m^3^ was 93.2%. Exposure levels above this threshold were distributed evenly across counties and seasons (Table 3: Cohort Characteristics Dichotomized by whether or not Exposed to Maximum of 35 ug/m^3^ in Pre‐Operative Period).

**TABLE 2:** Cohort characteristics dichotomized by presence of a complication.

Factor	Level	Overall	No	Yes	Rate	SMD
*n*		49,615	47,204	2411	4.85%	
Complications = Yes (%)		2411 (4.8)	0 (0.0)	2411 (100.0)		
Procedure year factor (%)	2016	15,375 (31.0)	14,586 (30.9)	789 (32.7)	5.13%	0.062
	2017	17,536 (35.3)	16,661 (35.3)	875 (36.3)	4.98%	
	2018	16,704 (33.7)	15,957 (33.8)	747 (31.0)	4.47%	
Elix (mean [SD])		0.89 (1.95)	0.6 (1.52)	5.15 (3.72)		1.576
Sex = Female (%)		25,911 (52.2)	24,909 (52.8)	1002 (41.6)	3.86%	0.226
Season (%)	None	17,044 (34.4)	16,207 (34.3)	837 (34.7)	4.91%	0.013
	Fire	16,472 (33.2)	15,667 (33.2)	805 (33.4)	4.88%	
	Cars	16,099 (32.4)	15,330 (32.5)	769 (31.9)	4.77%	
Disadvantage1317 (mean [SD])		0.06 (0.04)	0.06 (0.04)	0.07 (0.04)		0.218
Age (mean [SD])		53.22 (17.67)	53.11 (17.72)	55.28 (16.59)		0.126
County name (%)	Box Elder	672 (1.4)	621 (1.3)	51 (2.1)	7.58%	0.106
	Cache	1098 (2.2)	1041 (2.2)	57 (2.4)	5.19%	
	Davis	7047 (14.2)	6736 (14.3)	311 (12.9)	4.41%	
	Salt Lake	34,399 (69.3)	32,775 (69.4)	1624 (67.4)	4.72%	
	Utah	3847 (7.8)	3612 (7.7)	235 (9.7)	6.10%	
	Weber	2552 (5.1)	2419 (5.1)	133 (5.5)	5.21%	

*Note:* The complication rate fell slightly (*p* = 0.014) from 2016 to 2018, with an overall rate of 4.85%. The Elixhauser comorbidity score was marked higher for those with complications (5.15) than those without (0.6), while age only varied slightly by those without (53.11) and with (55.28) complications. Complication rates by County also varied, with Box Elder (7.58%) having the greatest rate and Davis with the lowest (4.41%).

**TABLE 3:** Cohort characteristics dichotomized by whether or not exposed to maximum of 35 ug/m^3^ in pre‐operative period.

Factor	Level	Overall	Max PM_2.5_ > 35	Max PM_2.5_ < 35	SMD
*n*		49,615	1147	48,468	
Complications (%)	No	47,204 (95.1)	1068 (93.1)	46,136 (95.2)	0.089
	Yes	2411 (4.9)	79 (6.9)	2332 (4.8)	
Procedure year factor (%)	2016	15,375 (31.0)	274 (23.9)	15,101 (31.2)	0.260
	2017	17,536 (35.3)	546 (47.6)	16,990 (35.1)	
	2018	16,704 (33.7)	327 (28.5)	16,377 (33.8)	
Elix (mean [SD])		0.89 (1.95)	1.02 (2.00)	0.88 (1.95)	0.067
Sex (%)	Male	23,704 (47.8)	549 (47.9)	23,155 (47.8)	0.002
	Female	25,911 (52.2)	598 (52.1)	25,313 (52.2)	
Season (%)	None	17,044 (34.4)	0 (0.0)	17,044 (35.2)	1.042
	Fire	16,472 (33.2)	578 (50.4)	15,894 (32.8)	
	Cars	16,099 (32.4)	569 (49.6)	15,530 (32.0)	
Disadvantage (mean [SD])		0.06 (0.04)	0.07 (0.04)	0.06 (0.04)	0.310
Age (mean [SD])		53.22 (17.67)	52.26 (17.80)	53.24 (17.67)	0.056
County name (%)	Box Elder	672 (1.4)	18 (1.6)	654 (1.3)	0.058
	Cache	1098 (2.2)	29 (2.5)	1069 (2.2)	
	Davis	7047 (14.2)	175 (15.3)	6872 (14.2)	
	Salt Lake	34,399 (69.3)	786 (68.5)	33,613 (69.4)	
	Utah	3847 (7.8)	77 (6.7)	3770 (7.8)	
	Weber	2552 (5.1)	62 (5.4)	2490 (5.1)	

*Note:* This table dichotomizes patients based on their exposure to any day of PM_2.5_ greater than 35 ug/m^3^ in the 7 day pre‐operative period. In this instance, the complication rate was higher when patients were exposed to PM_2.5_ > 35 (SMD = 0.089). Notably, 2017 had much higher exposure to elevated PM_2.5_ (546 cases, 47.6% of total exposures), while disadvantage was not dramatically different between higher (0.07) and lower (0.06). Finally, exposure to fire vs. cars as sources of pollution was nearly evenly split, indicating equal exposure to elevated inversion pollution and wildfire smoke.

### 3.3 Bayesian Multivariable Analysis

**FIGURE 3:**
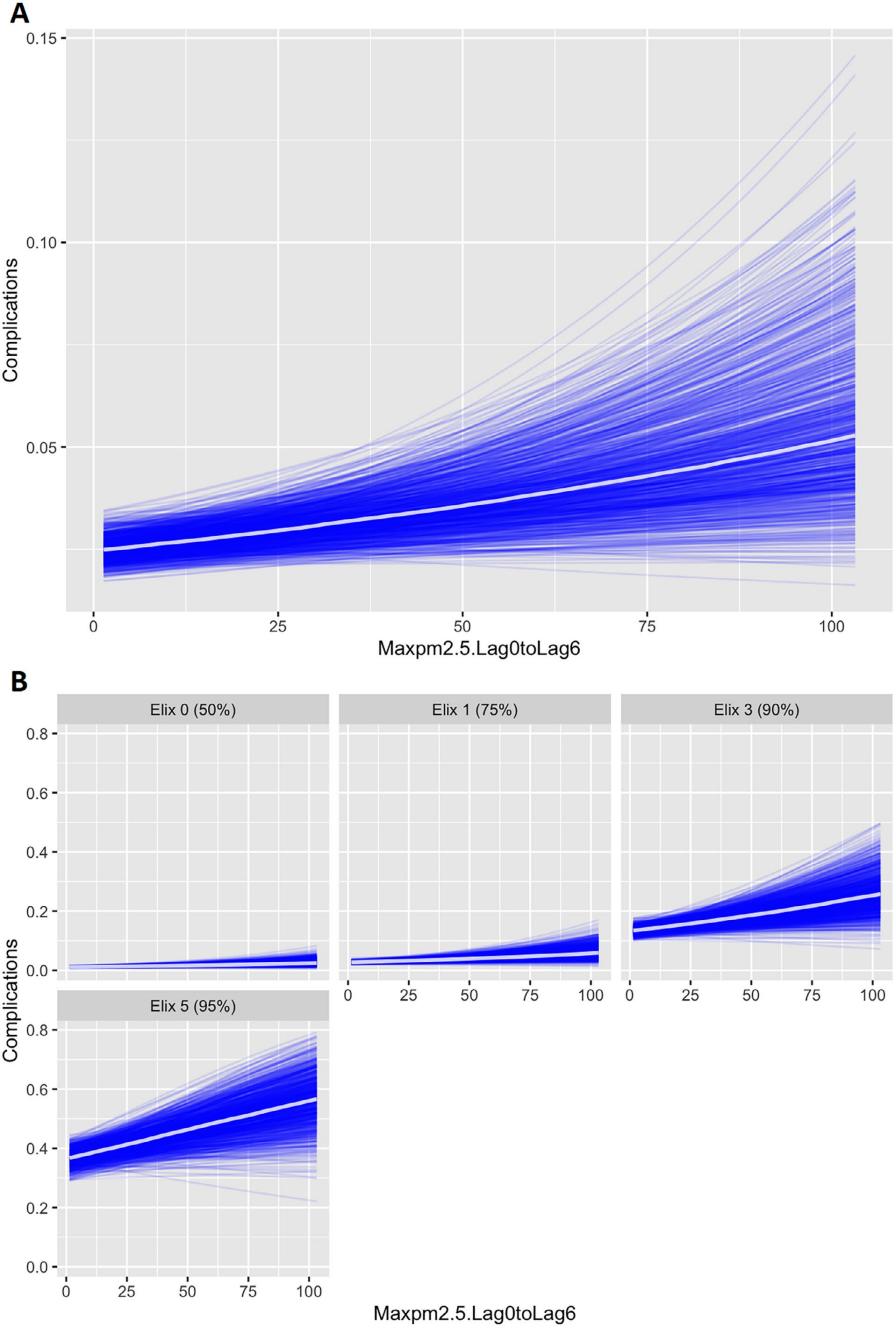
Maximum PM_2.5_ in the 7 days pre‐operatively (Lag0–Lag6) versus complication Rate. Panel (A) (top) details Complications Rate versus maximum PM_2.5_ of the 7‐day lag period, while Panel (B) (bottom) details the same broken down by Elixhauser Comorbidity Index. Complication rate is shown as absolute value, for example 0.04 equals a 4% complication rate. The PM_2.5_ values are the maximum observed in the 7‐day pre‐operative window, in ug/m^3^, Lag0 is day of surgery. In Panel (B) as noted, the overall complication rate for those with higher Elixhauser comorbidity index is higher at baseline, but rose more quickly with elevations in PM_2.5_. This response to PM_2.5_ continued to increase in magnitude as Elixhauser index increased. Note that the effect of PM_2.5_ was greater among those with Elixhauser of 5, but that the baseline complication rate was also higher.

In our main Bayesian multivariable model, increasing PM_2.5_ concentrations were associated with increased posterior probability of postoperative complications. The regression coefficient estimate was 0.01 (95% CrI: 0.00–0.01). Bayesian hypothesis testing showed a 99% posterior probability of increased complications with increasing PM_2.5_. The posterior log odds ratio (OR) was 1.008 per 1 ug/m^3^ increase and 1.082 per 10 ug/m^3^ increase in maximum preoperative PM_2.5_. Clinically, this corresponds to an ~8% increase in the chance of complication for every 10 ug/m^3^ increase in the maximum PM_2.5_ observed in the 7‐day preoperative period. An increase from 1 to 30 ug/m^3^ corresponded to a > 27% higher odds (95% CrI: 4%–55%), with a curvilinear exposure‐response pattern (Figure 3).

There was no apparent change in this association across seasons (fire, inversion, baseline), nor did adjustment for neighborhood disadvantage alter the findings. Effect sizes were greater among patients with higher comorbidity burden (Elixhauser ≥ 3 vs. < 1 respectively) as shown in Figure 3. Overall the parameters in the model demonstrated adequate fit and explained about 1/4th of the variance (*R* ^2^ = 0.28). Model estimation satisfied usual criteria (Gelman‐Rubin R‐hat, ESS, posterior predictive error checks). The posterior density plots for model parameters indicate reasonable unimodal distributions. Our sensitivity analysis exploring one weakly informative prior did not change the inferences or results. Our findings were robust to model parameters and model specifications. Details of the sensitivity analysis and model robustness are provided in the Supporting Information.

## 4 Discussion

In our single center cohort of over 49,000 patients undergoing elective surgery, higher preoperative PM_2.5_ exposure in the 7 days before surgery was associated with increased postoperative complication risk. The association was dose‐dependent, consistent across sensitivity analyses, and aligned with prior air pollution epidemiology literature [42]. The effect was most pronounced in patients with higher comorbidity burdens (Figure 3), underscoring the potential clinical relevance of acute exposures. However, this observational analysis does not identify causal effects of PM_2.5_, as PM_2.5_ should be interpreted as an exposure marker within complex air pollution mixtures rather than an isolated causal agent.

### 4.1 Methodological Contribution

The primary contribution of this study is methodological: we demonstrate how hierarchical Bayesian modeling can be applied to perioperative environmental epidemiology, thus offering probabilistic interpretability, regularization, and uncertainty quantification not directly available in conventional frequentist approaches. When examining single center clinical datasets in the context of perioperative environmental health studies, uncertainty quantification and protection against spurious associations become particularly important. Single‐center cohorts often involve correlated patient, procedural, and neighborhood‐level factors that can amplify statistical noise and complicate interpretation. In this setting, Bayesian hierarchical modeling provides a principled framework for stabilizing estimates, transparently characterizing uncertainty, and generating probabilistic effect summaries suitable for multicenter study planning.

Bayesian inference also provides direct probabilistic statements about risk while improving interpretability for clinical audiences, rather than indirect inferences about hypothetical repeated samples, as in frequentist methods, which are often misinterpreted as probabilities about the parameter itself. The Bayesian approach avoids this ambiguity, and thus enables transparent communication of uncertainty that aligns more naturally with clinical reasoning. Bayesian inference also facilitates assessment of sensitivity to prior assumptions: we compared models fit with both a standard Normal prior and an *R* ^2^ *D* ^2^ shrinkage prior, thereby finding consistent inferences across specifications. This robustness supports the stability of our findings and illustrates how Bayesian methods allow explicit testing of prior choices, and that our results were robust to these associations. By contrast, frequentist regression relies on implicit assumptions that are rarely interrogated directly, and inferences are often summarized only by point estimates and *p*‐values. The Bayesian approach, by making such assumptions explicit and allowing for their testing transparently as we did, facilitates clearer communication of risk to clinical audiences. Additionally, posterior probabilities (e.g., 99% probability of increased complications with rising PM_2.5_) and credible intervals provide more intuitive measures of uncertainty than *p*‐values. Our case study thus illustrates the feasibility and transparency of Bayesian methods for complex perioperative datasets, especially so in the context of single‐center preliminary data such as ours.

### 4.2 Comparison With Prior Work

Few other studies that have examined adverse impacts of air pollution on perioperative outcomes have been limited in scope, methodologies, and patient populations: A majority of these studies examined only organ transplants [20], with both kidney transplants and lung transplants susceptible to adverse outcomes with various pollution exposures [20, 43]. Recent data from China also found increased mortality after surgery, especially among surgical oncology patients with an overall higher adjusted 30‐day mortality per 10 μg/m^3^ increase in PM_2.5_ [17]. Recent data from California also suggest pediatric patients are susceptible to adverse pulmonary events under anesthesia during wildfire events [19]. Our results are broadly consistent with these findings, and this alignment supports the plausibility of the observed association in our cohort. Though our intent was not to establish definitive causal identification, our findings provide probabilistic associational evidence consistent with prior epidemiologic literature and motivate further multicenter investigations.

### 4.3 Limitations

Our study has several limitations. We estimated exposure based on census tract locations, without accounting for chronic exposures (e.g., highways, industrial sources), workplace, or indoor exposures. Additionally, we evaluated PM_2.5_ mass concentration and did not have data on particulate composition that may influence toxicity [44]. We further could not control for in‐home filtration or other personal mitigation measures, which may be less available in more socially vulnerable neighborhoods. We also lacked comprehensive race and ethnicity data in our Epic dataset, and our limited sample size prevented us from categorizing by surgical specialty. While our region of Northern Utah is predominately white with a large Hispanic minority, the metropolitan portion is close to the median diversity index for mid‐size US cities. Additionally, unmeasured confounding is also a possibility, as is incorrect inferences due to the ecological fallacy [45]. Finally, as a single‐center cohort with a composite outcome, generalizability is limited and replication in a larger, multi‐center cohort is essential to further our findings.

### 4.4 Implications and Conclusion

Our findings highlight the feasibility of Bayesian modeling in perioperative research as well as the potential to explore acute environmental exposures as modifiable perioperative risk factors. The approach demonstrated here may inform future multicenter studies and more refined outcome definitions. This approach can also be applied to more specific cohorts as sample sizes allow, and eventually inform predictive risk modeling in perioperative environmental epidemiology. For now, these results should be interpreted as exploratory, offering a proof‐of‐concept that Bayesian methods can enhance interpretability and transparency in environmental health research. While our findings overall are suggestive that fine particulate matter exposure may be a risk factor for adverse postoperative outcomes, our limited single‐center cohort should be interpreted as exploratory probabilistic evidence regarding an existing causal hypothesis. These results inform future multicenter investigations evaluating generalizability across diverse geographic and clinical settings.

## Author Contributions

J.F.P. conceptualized the study and drafted the content with revisions from M.J.S., N.W., T.M.B., N.L.P., C.K.K., M.A.H. C.K.J. and C.K.K. organized the datasets and provided them to N.L.P. for statistical analysis. N.L.P. performed the primary Bayesian modeling, with input and revisions from J.F.P., M.A.H., J.S.G., B.K.G. C.S.R., M.J.S. and T.M.B. provided critical feedback on the environmental exposure modeling and PM2.5 implications context. J.V.P. provided critical feedback and early figure revisions, while J.A.W. contributed to literature background. L.L. and N.W. provided geospatial analysis and patient geocoding, as well as critical feedback on exposure modeling. All authors critically revised the manuscript and take responsibility for the content.

## Funding

This study was funded from an intramural University of Utah Wilkes Center for Climate Science and Policy seed grant [to J.F.P and N.W] as well as partially supported by the National Institute of Health National Institute of Environmental Health Sciences and National Cancer Institute (NIH grant 5R01ES029528‐05 [to MS], 5R37CA276365‐02 [to N.W]). Further funding was from the National Science Foundation (NSF grant 2051246 [BG] and 2153019 [B.G]). The funding sources were not involved in the study design, collection, analysis, and interpretation of the data, in writing the report, or in the decision to submit the article for publication. The content is solely the responsibility of the authors and does not necessarily represent the official views of the National Institutes of Health, the National Science Foundation, or the University of Utah Wilkes Center for Climate Science and Policy.

## Disclosure

All work was done at the Salt Lake City campus of the University of Utah on secure servers, with remote access granted to outside collaborators.

## Ethics Statement

The study was approved as Exemption Category 4 by the Institutional Review Board at the University of Utah, with IRB approval number 00142167. Prior to access of data, we wrote of our intention to analyze the influence of environmental factors and air pollution on postoperative outcomes with the University of Utah IRB. All authors have seen and approved the manuscript.

## Conflicts of Interest

Ben Goodrich and Jonah Gabry are both member‐managers of G.G. Statistics LLC. Other authors declare no conflicts of interest.

## Supporting information


**Data S1:** aas70235‐sup‐0001‐Supinfo.docx.

## Data Availability

The data that support the findings of this study are available from the corresponding author upon reasonable request.
