# Supplementary material for: Bayesian Analysis of Postoperative Complication Risk Associated With Preoperative Exposure to Fine Particulate Matter: A Single‐Center Cohort Study
Source: Acta Anaesthesiol Scand. 2026 Apr 26;70:e70235. doi: 10.1111/aas.70235 (PMC13111187; doi:10.1111/aas.70235)
Supplement: Supplementary file 1 — Data S1: aas70235‐sup‐0001‐Supinfo.docx. [file AAS-70-0-s001.docx]

**Supplemental Appendix : Full Model Specification, Sensitivity Model Specification, and Outputs**

This appendix provides the complete mathematical specification, prior distributions, and posterior summaries for the primary analytic model.

**S1. Model Specification**

Let Complications denote whether patient experienced any postoperative complication.

Model Formula = Complications ~ Sex + ProcedureYearFactor + s(Age, Elix) + Maxpm2.5.Lag0toLag6 + Season + Disadvantage1317 + (1 | Tract2010)

Sex, procedure year, and season were modeled as categorical fixed effects.
PM2.5 and Neighborhood Disadvantage were modeled as linear effects.
(Age, Elix) was a 2-dimensional isotropic thin plate spline with k = 30 knots to allow for flexible, potentially non-linear and interactive effects.

Tract2010 is a random intercept for census tract.

Priors:

Regression coefficients: normal(mean=0,SD=1)
Random-effect and spline SDs: normal(mean=0,SD=1)

Intercept: Student-t(degrees of freedom=3,mean=0,SD=2.5)

Models fit using Hamiltonian Monte Carlo (6 chains, 2000 iterations, 1000 warmup).

**S2. Model Output**

summary(fit1004.tract.pm2.5.brms, priors = TRUE)

Family: bernoulli

Links: mu = logit

Formula: Complications ~ Sex + ProcedureYearFactor + s(Age, Elix) + Maxpm2.5.Lag0toLag6 + Season + Disadvantage1317 + (1 | Tract2010)

Data: tmp.df (Number of observations: 49615)

Draws: 6 chains, each with iter = 2000; warmup = 1000; thin = 1;

total post-warmup draws = 6000

Priors:

b ~ normal(0, 1)

Intercept ~ student_t(3, 0, 2.5)

<lower=0> sd ~ normal(0, 1)

<lower=0> sds ~ normal(0, 1)

Smoothing Spline Hyperparameters:

Estimate Est.Error l-95% CI u-95% CI Rhat Bulk_ESS Tail_ESS

sds(sAgeElix_1) 4.61 0.43 3.82 5.49 1.00 2604 3545

Multilevel Hyperparameters:

~Tract2010 (Number of levels: 476)

Estimate Est.Error l-95% CI u-95% CI Rhat Bulk_ESS Tail_ESS

sd(Intercept) 0.43 0.04 0.36 0.51 1.00 2067 3482

Regression Coefficients:

Estimate Est.Error l-95% CI u-95% CI Rhat Bulk_ESS Tail_ESS

Intercept -3.75 0.09 -3.93 -3.58 1.00 5993 4829

SexFemale -0.22 0.05 -0.31 -0.12 1.00 9361 4343

ProcedureYearFactor2017 -0.23 0.06 -0.35 -0.11 1.00 11636 4654

ProcedureYearFactor2018 -0.33 0.06 -0.45 -0.20 1.00 8575 4407

Maxpm2.5.Lag0toLag6 0.01 0.00 0.00 0.02 1.00 10278 4392

SeasonFire -0.20 0.06 -0.33 -0.07 1.00 9480 4265

SeasonCars -0.19 0.07 -0.34 -0.05 1.00 10231 4267

Disadvantage1317 1.46 0.85 -0.22 3.14 1.00 4223 4581

sAgeElix_1 -2.68 0.91 -4.45 -0.88 1.00 8122 4426

sAgeElix_2 9.59 0.55 8.52 10.67 1.00 5779 4384

Draws were sampled using sample(hmc). For each parameter, Bulk_ESS

and Tail_ESS are effective sample size measures, and Rhat is the potential

scale reduction factor on split chains (at convergence, Rhat = 1).

**Well calibrated in the highest-risk decile**
**Reasonably calibrated in mid-range deciles**
**Slightly overpredicting risk in lower deciles**

decile mean_pred mean_obs n

*<int>* *<dbl>* *<dbl>* *<int>*

1 1 0.00611 0.00262 4962

2 2 0.00782 0.00363 4962

3 3 0.00907 0.00464 4962

4 4 0.0103 0.00846 4962

5 5 0.0118 0.00806 4962

6 6 0.0136 0.0111 4961

7 7 0.0166 0.0123 4961

8 8 0.0238 0.0256 4961

9 9 0.0556 0.0712 4961

10 10 0.3310 0.3380 4961


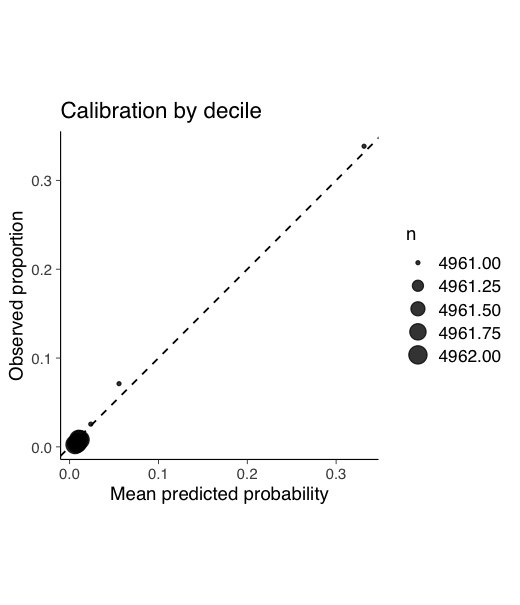


**S3. Sensitivity Model Specification**

Let Complications denote whether patient experienced any postoperative complication.

Model Formula = Complications ~ Sex + ProcedureYearFactor + s(Age, Elix) + Maxpm2.5.Lag0toLag6 + Season + Disadvantage1317 + (1 | Tract2010)

Sex, procedure year, and season were modeled as categorical fixed effects.
PM2.5 and Neighborhood Disadvantage were modeled as linear effects.
(Age, Elix) was a 2-dimensional isotropic thin plate spline with k = 30 knots to allow for flexible, potentially non-linear and interactive effects.

Tract2010 is a random intercept for census tract.

Priors:

Regression coefficients: R2D2M2(mean R2=0.25,precision=4, D=0.5)
Random-effect and spline SDs: R2D2M2(mean R2=0.25,precision=4, D=0.5)

Intercept: Student-t(degrees of freedom=3,mean=0,SD=2.5)

**S4. Sensitivity Model Output**

summary(fit1004a.tract.pm2.5.brms, priors = TRUE)

Family: bernoulli

Links: mu = logit

Formula: Complications ~ Sex + ProcedureYearFactor + s(Age, Elix) + Maxpm2.5.Lag0toLag6 + Season + Disadvantage1317 + (1 | Tract2010)

Data: tmp.df (Number of observations: 49615)

Draws: 6 chains, each with iter = 2000; warmup = 1000; thin = 1;

total post-warmup draws = 6000

Priors:

b ~ R2D2(mean_R2 = 0.25, prec_R2 = 4, cons_D2 = 0.5, autoscale = TRUE, main = TRUE)

Intercept ~ student_t(3, 0, 2.5)

<lower=0> sd ~ R2D2()

<lower=0> sds ~ R2D2()

Smoothing Spline Hyperparameters:

Estimate Est.Error l-95% CI u-95% CI Rhat Bulk_ESS Tail_ESS

sds(sAgeElix_1) 6.13 0.83 4.69 7.92 1.00 1974 2577

Multilevel Hyperparameters:

~Tract2010 (Number of levels: 476)

Estimate Est.Error l-95% CI u-95% CI Rhat Bulk_ESS Tail_ESS

sd(Intercept) 0.44 0.04 0.36 0.52 1.00 2093 3665

Regression Coefficients:

Estimate Est.Error l-95% CI u-95% CI Rhat Bulk_ESS Tail_ESS

Intercept -3.81 0.09 -3.99 -3.64 1.00 4141 5318

SexFemale -0.21 0.05 -0.31 -0.11 1.00 5430 3707

ProcedureYearFactor2017 -0.22 0.06 -0.34 -0.10 1.00 4352 5485

ProcedureYearFactor2018 -0.33 0.06 -0.45 -0.20 1.00 5431 4829

Maxpm2.5.Lag0toLag6 0.01 0.00 0.00 0.01 1.00 4113 3897

SeasonFire -0.18 0.07 -0.31 -0.05 1.00 4149 4187

SeasonCars -0.18 0.08 -0.32 -0.03 1.00 4504 2855

Disadvantage1317 1.26 0.86 -0.33 2.96 1.00 3665 4107

sAgeElix_1 14.22 0.74 12.80 15.71 1.00 8277 5150

sAgeElix_2 0.49 1.67 -2.58 4.58 1.00 5896 4247

Draws were sampled using sample(hmc). For each parameter, Bulk_ESS

and Tail_ESS are effective sample size measures, and Rhat is the potential

scale reduction factor on split chains (at convergence, Rhat = 1).

**S5. Model Comparison**

Leave-one-out cross validation showed no difference in predictive ability between the models using the expected log of the posterior predictive distribution (elpd).

loo_compare(fit1004.tract.pm2.5.brms.loo, fit1004a.tract.pm2.5.brms.loo)

elpd_diff se_diff

fit1004.tract.pm2.5.brms 0.0 0.0

fit1004a.tract.pm2.5.brms 0.0 0.2
